# Supplementary material for: Content-rich biological network constructed by mining PubMed abstracts
Source: BMC Bioinformatics. 2004 Oct 8;5:147. doi: 10.1186/1471-2105-5-147 (PMC528731; doi:10.1186/1471-2105-5-147)
Supplement: Additional File 2 — The original results of the above study (non-essential files are deleted to keep the file size under the limit set by BMC bioinformatics). [file 1471-2105-5-147-S2.bz2 › chilibotAdditionalFile2/dip05/305ID10428074E1302/html/APP_APOE.html]

 


 **APP** and **APOE** 
  
Found 392 abstracts in PubMed, retrieved 05.  
 

 What does Google say? 
 PDF only 
| .edu only 

---

**Interactive relationship** (e.g. stimulation, inhibition, etc)

**Non-interactive relationship** (e.g. studied together, co-existance, homology, etc.)

- The  **APOE** \*4P mutation was observed in heterozygous state in four subjects, two AD 1  [ **APP** ] .71% and two controls 1.65%.  Ref: 12815750 Am J Med Genet, 2003
- The present study was undertaken to test the hypothesis that estrogen can attenuate Ang II induced AAA  [ **APP** ]  in apolipoprotein E  [ **APOE** ]  deficient mice via its anti inflammatory mechanism.  Ref: 12855485 Arterioscler Thromb Vasc Biol< MedlineTA>Arterioscler Thromb Vasc Biol, 2003
